# Does migrant background predict to what extent colorectal cancer patients want to be informed about their life expectancy? – a cross-sectional analysis

**DOI:** 10.1186/s12939-019-1105-0

**Published:** 2019-12-05

**Authors:** Marja Leonhardt, Katja Aschenbrenner, Martin E. Kreis, Johannes C. Lauscher

**Affiliations:** 1 Department of General, Visceral and Vascular Surgery, Campus Benjamin Franklin, Charité University of Medicine Berlin, 12203 Berlin, Germany; 2 Faculty of Health Studies, VID – Specialized University, Mailbox 184 Vinderen, 0319 Oslo, Norway

**Keywords:** Migrant background, Colorectal cancer, Information, Life expectancy, Trust in the treating physician

## Abstract

**Background:**

Although migrant health is a topic of interest across Europe and although health care services in Germany consider migrant health issues, people with a migrant background often experience difficulties regarding health care provision. The prevalence of various cancers among migrants is lower relative to non-migrants although this equalizes with increasing duration of residence. There are documented differences in health behavior and disease-coping strategies between migrants and non-migrants, but data are scarce on this subject. This analysis investigates the extent of information migrant and non-migrant colorectal cancer (CRC) patients in Germany want about their life expectancy and the level of trust they have in their treating physician.

**Method:**

Data from 522 CRC patients were collected through a self-reported questionnaire. Migrant background was determined by the patients’ and/or their parents’ birthplace. Bivariate analyses were applied to determine the differences between migrants and non-migrants. A multivariate analysis was used to measure the effect of migration background, demographics, and cancer stage and treatment on the preferred extent of information about life expectancy and trust in their treating physician.

**Results:**

There were no significant differences regarding demographics or cancer stage and treatment between migrant and non-migrant CRC patients. Having a migrant background had no influence on the level of trust in the treating physician, but migrants preferred to be less informed about their life expectancy than non-migrants (21.4% vs. 13.4%, *p* = 0.04). The multivariate analysis showed that men (aOR = 2.102, CI: 1.123–3.932) and patients with a non-migrant background (aOR = 5.03, CI: 1.02–24.73) preferred receiving information about the approximate value of their life expectancy, rather than receiving no information.

**Conclusion:**

The study found more similarities than discrepancies between migrant and non-migrant CRC patients regarding demographic factors and stage of disease and treatment, which may be a consequence of an increasingly homogeneous cross-cultural society. However, cultural differences between the minority and host population remain and should always be taken into account in daily clinical practice and in the communication skills training of health care professionals. The study also indicates that recording migration background into health registers would facilitate migrant-sensitive research.

## Background

Conducted in 2017, the latest micro-population census counted 19.3 million individuals who either migrated themselves or had at least one parent who migrated to Germany. This accounts for 23.6% of the entire population in Germany [1]. The migrant population is heterogeneous in terms of cultural and religious background, socioeconomic status, language proficiency, literacy, and health behavior [2]. Even though health care services in Germany take migrant health issues into account, people with a migrant background often experience difficulties regarding health care provision [3]. Migrants have to overcome language barriers and cross-cultural challenges within health care, especially when it comes to noncommunicable diseases such as cancer [4]. CRC is the second most common cancer type in Europe [5]; in Germany, one in seven of all diagnosed cancers is located in the colorectum [6]. In Germany, all those with statutory health insurance (about 90% of the population) have the right to participate in free CRC screening programs starting from the age of 50, but migrants make less use of this service than the autochthonous population does [7, 8].

Furthermore, migrants attend follow-up consultations, which are offered to all CRC patients, less frequently than non-migrants do [9]. Migrant patients report language barriers, difficulty in understanding the local health system and a lack of cultural sensitivity and understanding from medical professionals as the reasons for this behavior [10, 11]. Comprehensible medical information that meets the needs of the individual is essential for every patient, regardless of the migrant background. Studies show [12] that sharing prognostic information with patients may facilitate a better understanding of their illness and greater patient involvement in medical decision making [13]. Furthermore, according to Hillen et al., oncologists gain their patients’ trust if they present themselves as competent, honest, and caring. Caring behavior—such as making emotionally supporting statements—has the strongest effect on trust [14]. Cultural sensitive communication on behalf of health professionals is needed to prevent misunderstandings and medical malpractice [15]. Although physicians are aware of the importance of cross-cultural care, especially when it comes to aspects of information delivery, there is a gap between physicians’ willingness to deliver cross-cultural care and the willingness in other clinical and technical areas [16]. To the best of our knowledge, variances in the preferences for exact information regarding prognosis and the level of trust in the treating physician between migrants and non-migrants have not yet been explored. Hence, in the current study, we aimed to explore the differences in migrant and non-migrant CRC patients’ preferences for information about life expectancy and level of trust in the treating physician. According to the German Public Health Institute (Robert Koch Institute), data on migrant health and behavior in Germany are still scarce [17]. The findings may contribute to establishing equal and appropriate treatment and care plans for both migrant and non-migrant CRC patients and promote cross-cultural knowledge for health professionals which might minimize the gap between willingness of delivering cross-cultural care and performance in other clinical areas.

## Methods

### Data source and procedure

**Fig. 1:**
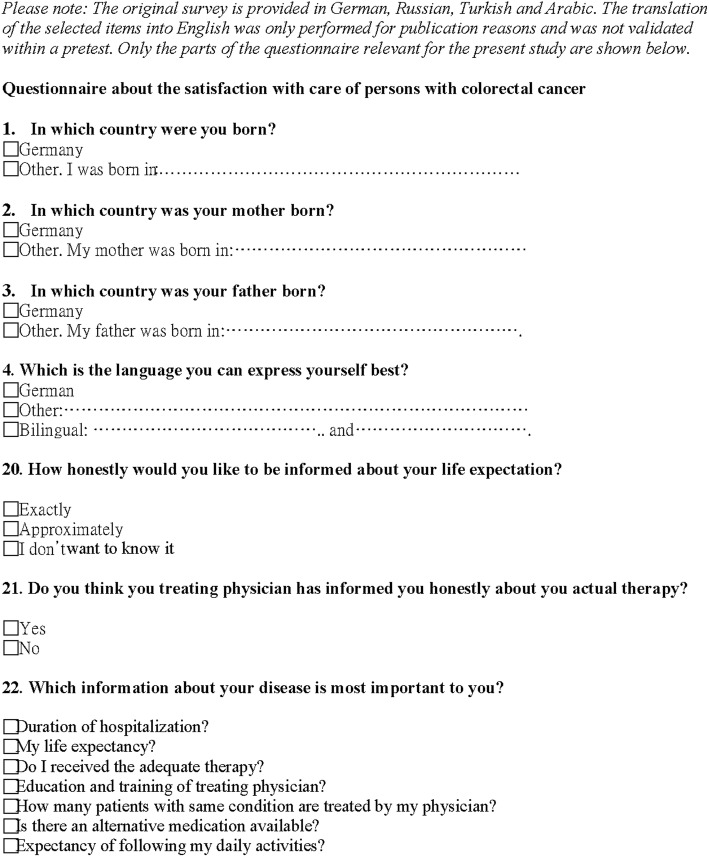
Questionnaire items used for analysis

The present article is part of a larger survey conducted between March and August 2015 that explored the characteristics and potential disparities of non-migrant and migrant CRC patients regarding their satisfaction and subjective perceptions of care [9]. The survey applied an established and standardized questionnaire (EXPRESSION) that was used in studies on patient-physician-relationships, patients’ expectations and perceptions of breast, peritoneal and ovarian cancer [18, 19] and that was adapted for CRC patients. The adapted questionnaire was pretested at the Charité University of Medicine (Campus Benjamin Franklin) and then translated from German into Turkish, Arabic, and Russian by certified interpreters to increase the participation rate of non-German native speakers. The selection of the languages was based on the most frequently spoken languages in the city of Berlin [20]. Because the primary objective of the main survey was to explore the possible differences between migrant and non-migrant CRC patients, not to develop a questionnaire, we did not assess the validity and reliability of the adapted survey items as the original items have been validated earlier. More information on the applied questionnaire and the recruitment of the study population has been published elsewhere [9]. Figure 1 shows all of the items from the questionnaire used in the current analysis. Patients who were registered between 2004 and 2014 in the prospectively kept CRC database of the Charité Comprehensive Cancer Centre (CCCC) and were still alive in 2015 were invited to fill out the adapted questionnaire. The dataset that was obtained from this survey was supplemented with relevant clinical parameters (cancer stage, received treatment, and biomedical and demographic information) from the CCCC database.

### Sample and procedure

**Fig. 2:**
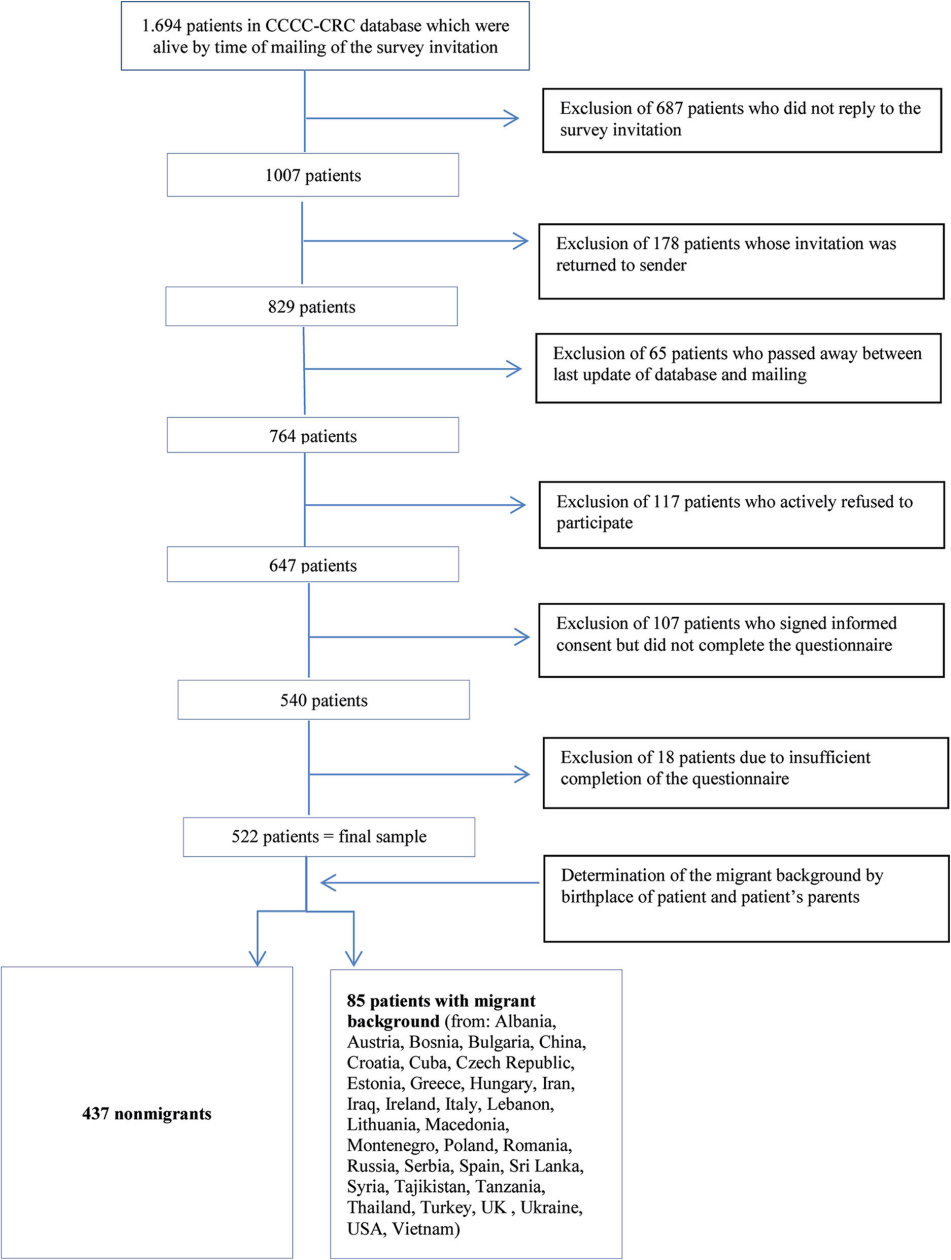
Flowchart of the data inclusion process

The survey focused on patients with CRC (ICD 10 C18-C20: Malignant neoplasm of the colon, the recto-sigmoid junction, and the rectum) who were registered between 2004 and 2014 in the CCCC database, irrespectively of being in a treatment or having completed a treatment by the time the survey was conducted. Patients were included in the present analysis if they were older than 18 years and completed the survey questionnaire in 2015. The present article focused on patients’ (clinical) characteristics and items regarding their preferred information and trust in their treating physician. Data from 522 patients were used for the final analysis. The data inclusion process is presented in Fig. 2.

The ethics committee of the medical faculty at Charité University Hospital Berlin approved all study procedures (EA4/131/14). All study participants signed an informed consent form.

### Measures

#### Outcome and grouping variables

In the analysis, we compared patients with a migrant background (migrants) and native Germans (non-migrants). We categorized the patients who had migrated to Germany themselves or had at least one parent who had migrated to Germany as having a migrant background. This is done according to the practice in comparable studies [21, 22] and in line with the definition from the Federal Office for Migration and Refugees for an individual with a migrant background [2]. Patients with a migrant background were categorized additionally as first- and second-generation migrants according to their own and their parents’ birthplace. For better readability, the term ‘patient with a migrant background’ is used synonymously with ‘migrant’ if not otherwise stated.

The outcome of interest was the extent of information favored about life expectancy; this was used as the dependent variable in the bi- and multivariate analyses. The patients were asked, “How precisely would you like to be informed about your life expectancy?” and were given the answer categories “exactly,” “approximately,” and “I don’t want to know,” as well as “Do you think that your treating physician was honest with you about your treatment plan?” with the answers “Yes” or “No.”

#### Independent variables

Apart from the demographic variables, cancer stage (UICC stage), and the received treatment were used in the bi- and/or multivariate analyses. Additionally, the questionnaire item “Which information about your disease is the most important to you?”, the German Procedure Classification (OPS) and family history of CRC were used as independent variables in the descriptive statistics. Because it is assumed that the ability of expressing oneself in the host country’s language has an influence on the choice of treatment and the comprehension of any informed conversation between the patient and the physician [23], the patient’s best-spoken language was used as a further independent variable. The survey patients were asked in which language they could express themselves best (German/other than German/bilingual). This variable is described as the best-spoken language and was computed dichotomously (German and German + another language/other than German) because every patient who stated bilingual included German as their first or second language.

### Statistical analysis

Non-migrants and migrants were compared regarding the independent variables; this was done using a chi-square test and Fisher’s exact test, depending on the variables’ scale. The summary statistics were calculated for all variables overall and by migrant background. The effect of migrant background on the preference regarding the precision of life expectancy was assessed by multinomial regression and binary logistic regression models, unadjusted (model 1) and adjusted for the covariates gender, age, UICC stage, cancer treatment, and best-spoken language as possible confounders (model 2). Unadjusted odds ratio (OR), adjusted odds ratio (aOR), and 95% confidence intervals (CI) were also calculated. “I don’t want to know my life expectancy” and “Yes, my treating physician was honest about the treatment plan,” respectively, were used as the reference categories.

Statistical significance was set at *p* < 0.05. Patient data were pseudonymized, and the analysis was performed using IBM SPSS Statistics for Windows, Version 25.0. Armonk, NY.

## Results

### Sample characteristics

Table 1 presents the description of the sample. Our study population comprised 522 CRC patients, of which 85 patients (16.3%) had a migrant background and originated from 34 different countries. Thereof, a higher portion (71.8%) was first-generation migrants. The 437 non-migrant patients were of the same ethnicity. The majority of the participants were male (57.7% in the group of non-migrants and 63.5% in the group of migrants, *p* = 0.315) and 60 to 69 years old (33% in the group of non-migrants and 28.7% within the group of migrants, *p* = 0.089). Most of the migrants (37.6%) specified that they could express themselves equally well in German and in another language, whereas 33% stated German was the language in which they could express themselves best, and 0.5% of the non-migrants and 29.4% of the migrants indicated a language other than German as their best-spoken language. Overall, best-spoken language was strongly associated with migrant background (*p* < 0.001). A UICC stage 0–II was registered by the time of diagnosis with the larger portion of the total sample (55.4% in non-migrants and 54.8% in migrants, *p* = 0.905). In the CRC patients, 3.5% of the migrants and 3.7% of the non-migrants (*p* = 0.997) had a family history of CRC. The most frequent surgical procedure in both groups was hemicolectomy (47.6% vs. 47.7%), followed by rectum resection with sphincter preservation (34.2% vs. 36.9%), *p* = 0.854. About 37% of the patients, both migrants and non-migrants, received chemotherapy, 20% received radiotherapy, and 16.7% of the non-migrants and 17.6% of migrants were treated with RCT. There were no significant differences between non-migrants and migrants regarding cancer treatment.

**Table 1:** Description of the study sample by migrant background (total sample: *n* = 522)

	Non-migrant background n total = 437		Migrant background n total = 85		*p*-value
	*n*	%	*n*	%	
Age					0.089*
< 50 years	54	12.4	17	20.0	
50–59 years	86	19.7	22	25.9	
60–69 years	144	33.0	24	28.2	
70–79 years	130	29.6	21	24.7	
≥ 80 years	23	5.3	1	1.2	
Gender					0.315*
Male	252	57.7	54	63.5	
Female	185	42.3	31	36.5	
Generation					§
German	437	100	0	0	
2nd generation	0	0	24	28.2	
1st generation	0	0	61	71.8	
Best spoken language					< 0.001*
German	435	99.5	28	33	
German and other language	0	0	32	37.6	
Other than German	2	0.5	25	29.4	
UICC stage ^a^					0.905^#^
UICC 0-II	240	55.4	46	54.8	
UICC III-IV	193	44.6	38	45.2	
Positive family history for CRC					0.997*
Unknown	52	11.9	10	11.8	
Yes	16	3.7	3	3.5	
No	369	84.4	72	84.7	
Chemotherapy					1.000^#^
No	273	62.5	53	62.4	
Yes	164	37.5	32	37.6	
Radiotherapy					0.764*
No	355	81.2	68	80	
Yes	82	18.8	17	20	
RCT					0.874^#^
No	364	83.3	70	82.4	
Yes	73	16.7	15	17.6	
German Procedure Classification (OPS)^b^					0.854*
Hemicolectomy	160	47.6	31	47.7	
Colectomy and proctocolectomy	12	3.6	2	3.1	
Extended colon resection with resection of neighboring organs	27	8.0	6	9.2	
Rectum resection with sphincter preservation	115	34.2	24	36.9	
Rectum excision without sphincter conservation	22	6.5	2	3.1	
Favored extent of information about life expectancy^c^					0.041*
Exactly	260	62.1	54	64.3	
Approximately	103	24.6	12	14.3	
I don’t want to know it	56	13.4	18	21.4	
Trust in the treating physician reg. Information about actual treatment plan^d^					0.562*
Yes	365	89	78	91.8	
No	45	11	7	8.2	
Most important information^e^					0.728*
Duration of hospitalization	39	10.4	8	10.8	
Life expectancy	72	19.2	14	18.9	
Adequate treatment received	81	21.6	14	18.9	
Training of treating physician	6	1.6	1	1.4	
No. of patients with same condition treated by physician	9	2.4	1	1.4	
Alternative medication	15	4.0	0	0	
Expectancy of following normal daily activities	14	3.7	3	4.1	
More than one answer given	139	37.1	33	44.5	

* chi-square test, ^#^ fisher s exact test, ^§^ p-value not applicable, ^a^ *N* = 517, ^b^ *N* = 401, ^c^ *N* = 503, ^d^ *N* = 495, ^e^ *N* = 449

When it came to the most important information regarding the patients’ disease, the statistical analyses showed no significant differences between the study groups. Most of the patients ticked more than one topic as being the most important. The non-migrants specified receiving adequate treatment (21.6%) and information about life expectancy (19.2%) as the second and third most important information, followed by information on the duration of hospitalization (10.4%). This applied also to the patients with a migrant background (18.9% for both “adequate treatment received” and “life expectancy”) and 10.8% for “duration of hospitalization.”

### Information about life expectancy

The descriptive statistics suggested that a higher proportion of CRC patients with a migrant background (21.4% vs. 13.4%; *p* = 0.041) did not want to be informed about their life expectancy whereas the majority in both groups (64.3% migrants; 62.1% non-migrants) wanted to know it exactly. Regarding the outcome, the descriptive statistics regarding a favored precision about life expectancy corresponded to a crude OR of 2.76 (CI: 1.24–6.14, *p* = 0.013). After adjusting for possible confounders in the second model, migrant background continued to have a significant effect on how precisely the patients wanted to be informed about their life expectancy, which is shown in Table 2. When controlling for gender, age, UICC stage, treatment, and best-spoken language, the desire to be approximately informed about life expectancy occurred 5.03 times (CI: 1.02–24.73, *p* = 0.047) more frequently when the patient was a non-migrant compared with those not wanting to know their life expectancy. Similarly, male CRC patients were more likely than females to prefer approximate information about their life expectancy (aOR = 2.10, CI: 1.23–3.92, *p* = 0.014). Furthermore, the findings show that it was more likely for patients with a less-advanced UICC stage (0–II) to prefer approximate information about life expectancy compared with those not wanting to know their life expectancy (aOR = 2.07, CI: 1.05–4.06, *p* = 0.036).

**Table 2:** Results of the multinomial regression model with “Favored extent of information about life expectancy” as a dependent variable

		Exactly ^a^			Approximately ^a^		
Independent variable		aOR	95%-CI	*p*-value	aOR	95%-CI	*p*-value
Migrant background (ref.: Migrant)	Non-migrant	1.137	0.399–3.240	0.811	5.031*	1.024–24.731	0.047
Gender (ref.: Female)	Male	1.586	0.930–2.705	0.090	2.102*	1.123–3.932	0.014
Age (ref.: ≥ 80 years)	< 50 years 50–59 years	2.412 2.016	0.577–10.088 0.531–7.648	0.228 0.303	2.621 1.204	0.532–12.916 0.264–5.490	0.217 0.673
	60–69 years	1.379	0.389–4.894	0.619	0.881	0.209–3.712	0.980
	70–79 years	0.987	0.287–3.399	0.984	0.715	0.175–2.914	0.805
Best spoken language (ref.: Other)	German/bilingual	2.043	0.637–6.555	0.230	0.600	0.111–3.253	0.568
UICC stage (ref.: III-IV)	0-II	1.488	0.838–2.642	0.175	2.065*	1.049–4.064	0.036
Chemotherapy (ref.: Yes)	No	0.771	0.371–1.599	0.484	0.531	0.227–1.241	0.144
Radiotherapy (ref.: Yes)	No	1.398	0.254–7.701	0.700	0.589	0.098–3.558	0.564
RCT (ref.: Yes)	No	0.596	0.085–4.183	0.602	2.234	0.273–18.289	0.454

^a^Reference group: “Don’t want to know”. * *p* < 0.05, Cox & Snell R^2^ = 0.06, Nagelkerke R^2^ = 0.07

Adjusted odds ratios (aOR) and 95% confidence intervals (95%-CI), *n* = 498

### Trust in the physician

Likewise, most of the study patients trusted their treating physician concerning the information about the treatment. Neither migrant background nor proficiency in the German language significantly influenced patients’ trust in their physician regarding honesty about treatment. When adjusting for possible confounders, the significance regarding these variables did not change, but an age of 70–79 seemed to have an effect; patients in this age group were more likely to trust their treating physician (4.22, CI: 1.13–15.85, *p* = 0.033). When focusing only on this age group, there were no significant differences between migrants and non-migrants (Chi^2^ [1]=2.51, *p* = 0.113). The results of model 2 (adjusted ORs) are presented in Table 3**.**

**Table 3:** Results of the logistic regression model with “Honesty of the treating physician about therapy” as dependent variable (reference: Yes)

Independent variable		aOR	95%-CI	p-value
Migrant background (ref.: Non-migrant)	Migrant	0.981	0.287–3.349	0.975
Gender (ref.: Male)	Female	1.126	0.614–2.061	0.701
Age (ref.: < 50 years)	50–59 years	1.464	0.390–5.489	0.572
	60–69 years	1.838	0.510–6.630	0.352
	70–79 years	4.220*	1.124–15.845	0.033
	≥ 80 years	2.648	0.746–9.401	0.132
Best spoken language (ref.: German/bilingual)	Other	0.395	0.076–2.039	0.267
UICC stage (ref.: 0-II)	III-IV	1.303	0.681–2.492	0.424
Chemotherapy (ref.: No)	Yes	1.423	0.661–3.060	0.367
Radiotherapy (ref.: No)	Yes	1.240	0.148–10.426	0.843
RCT (ref.: No)	Yes	0.506	0.048–5.301	0.569

Adjusted odds ratio (aOR) and 95% confidence intervals (95%-CI), *n* = 490

Cox & Snell R^2^ = 0.03, Nagelkerke R^2^ = 0.06, * p < 0.05

## Discussion

We compared CRC patient characteristics between migrants and non-migrants and examined which parameters influenced how precisely CRC patients wanted to be informed about their further life expectancy. We observed that having a migrant background and gender predicted the extent of clinical information favored regarding life expectancy. To the best of our knowledge, this is the first study on the desired precision of information concerning life expectancy, focusing on migrants.

In general, the fact that there were no differences regarding the demographic factors, UICC stage, and cancer treatment between migrant and non-migrant CRC patients may be understood as CRC patients—regardless of their ethnicity—had a similar cancer stage at the time of diagnosis, were treated equally, and received the same treatment choices, respectively. This might indicate that there are more similarities than discrepancies between CRC patients of different origins than assumed from the results of other migrant-specific studies that determined differences in terms of tumor biology, diagnostics, and treatment in breast cancer patients [24] or in health transition patterns [21]. Our results could be explained by the “healthy migrant effect,” which is the phenomenon that the overall health of migrants is often better than those who stay in their country of origin, possibly as a result of selective migration [22]. However, most of the migrants in our study population were first-generation migrants who, according to their age and language proficiency, migrated to Germany during the guest worker recruitment phase in the 1970s and 1980s. Hence, they may have adapted to the German lifestyle and diet by now, and the “healthy migrant effect” would not apply to them.

The fact that our study did not exactly identify the most important information for CRC patients might be because of the item construction: Patients were asked, “What information about your disease is most important to you?” and were given seven answer categories with more than one answer possible. This might have caused the majority of participants to mark more than one box, which impaired the statistical analysis. Our analysis revealed that there were no differences between migrants and non-migrants regarding the most important information for CRC patients.

Concerning the favored extent of information about life expectancy, the bivariate analysis showed that fewer patients with a migrant background wanted to be informed about their life expectancy compared with native German patients. In some cultures, cancer as a disease means fate, and it is believed that such a diagnosis implies death. One is powerless, and help is delegated to the healer: the medical experts [25]. Furthermore, a severe disease such as cancer underlies certain taboos in some cultures. Giving “strangers” an insight into family matters or one’s feelings and emotions can be very uncomfortable [26]. This would explain our findings of the multinomial regression model that migrant background had an influence on how precisely the CRC patient wanted to be informed. Compared with “I don’t want to know my life expectancy” as a reference category, non-migrants wanted to be approximately informed about life expectancy more often than migrants did. Indeed, the migrant study population originated from 34 countries, the majority of which are dominated by an Orthodox or Muslim religion. Tayjeb et al. found that Muslims tend to value spiritual and emotional support and believe that death is closely linked to fate [27]. Orthodox patients tend to interpret the end-of-life situation as given by God [28]. Thus, patients from cultures in which religion plays an important role possibly deal with death and estimated life expectancy in a less rational way than native Germans do. Still, our results should not be overemphasized because the majority of the study population wanted to be exactly informed about their life expectancy. Furthermore, the questionnaire did not assess religious affiliation, so we can only assume religious affiliation from the country of origin.

Another point that should be taken into account is the individualism–collectivism construct [29] that provides a framework for understanding cultural variations in communication. Germany is stated as an individualistic culture, which is more assertive and more direct in their conversation than Africans or Asians, who are collectivist. The latter have rather general situations and paralinguistic signals in mind, whereas individualist cultures turn their attention to the concrete statement and are more likely to omit the paralinguistic signals [30]. Interactions between individualist and collectivist cultures may have an impact on the extent of information wanted during a medical consultation. However, because the migrant population in our study comprised 34 countries of origin from both collectivist and individualist cultures, it would be problematic to draw strong conclusions according to this construct.

Other studies have shown that health care professionals and patients often report challenges in their interactions because of discrepancies in language [31, 32]. Our results show that language proficiency has no influence on how accurately patients want to be informed about their life expectancy. One could assume that being fluent in German may facilitate a better understanding of the medical consultation, which also deals with life expectancy. Being confronted with a negative message - which is often the case regarding the life expectancy of cancer patients - may be more easily understood in one’s mother tongue. On the other hand, we do not know whether the treating physician might have had proficiency in the language of the patient. Ilkilic reported that when it comes to end-of-live conversations between medical persons and patients, it is important to be aware of cultural traditions and to call in a professional translator to avoid misunderstandings. Making use of a relative as a translator may lead to misinterpretations [33]. This underlines the importance of language within the patient–physician relationship. A qualitative study approach, such as semi structured interviews, might be used in future research to clarify this issue. However, the majority of patients, both migrants and non-migrants, believed that their treating physician was honest with them about their treatment plan. Hence, this may be interpreted as equal treatment of all patients, regardless of migrant background.

Some limitations must be considered. Because of the retrospective assessment of migrant background, the migrant sample (16.3%) was quite small, which did not allow for stratification for language and country of origin, hence reducing the scope of interpretation when it came to the multivariate analysis. Nevertheless, the response rate of the patients with a migration background is comparable with other German migrant specific studies [34, 35]. Further, the term “person with a migrant background” includes many aspects, such as cultural and religious beliefs, language, health literacy, and duration of stay in the host country. What is more, familial culture was not assessed; there might be a difference between migrants living in Germany, those keeping up the traditions of the country of origin, and those cultivating the traditions of their host country. These aspects might influence the extent of information the individual patient desires. Besides language, we could not address these diversity factors in our analysis. In addition, it should be mentioned that the current study not only included those of non-German nationality, but also patients with a migrant background. Because most of the health registers and nationwide surveys in Germany do not assess the migrant background of an individual, migrant-specific health research is hindered and remains scarce. What is more, due to data protection regulations, it was impossible to merge our dataset with health service utilization data of the statutory health insurance system, which would have provided more comprehensive information of health behavior and attitudes.

This study provides some implications for clinicians who might work with migrant patients in their daily practice, e.g. delivering the information about life expectancy in a cultural sensitive way, as migrants tend do not want to know it exactly. However, our study highlights that migrant health research in Germany is hindered due to limited data availability. The assessment of nationality and/or migration background in health registers is not consistently established [36]. For example, the Cancer Register of Berlin, Brandenburg, Mecklenburg-West Pomerania, Saxony-Anhalt, Saxony and Thuringia decided in 2018 to eliminate the variable “nationality” from their register. Taking migrant health into account, registers in Germany should assess the migrant background according to the definition of the Federal Office for Migration and Refugees [2] which would facilitate migrant sensitive research.

## Conclusion

Our study found more similarities than discrepancies between migrant and non-migrant CRC patients than expected as far as patients’ characteristics and trust in the treating physician are concerned. Only the desired extent of information about life expectancy differed slightly between both groups. Our results could be interpreted as the consequence of a positive development toward greater equality within a cross-cultural society. Nevertheless, to avoid health disparities between migrant and non-migrant CRC patients and to ensure the best possible treatment for every patient, health care professionals should still be aware of the possible cultural differences between the minority and host populations. Knowledge of the culture in its complexity and awareness of the risk of stereotyping is a requirement for communicating with cancer patients in culturally sensitive ways. Health professionals should acknowledge potential differences in health values, basing these on the awareness that different cultures may influence each other. Within medical, and especially oncology training, cross-cultural competence and communication skills should be taught. Further policy implications are targeted at health registers, which ought to implement a common method to assess migration background to facilitate migrant sensitive health research.

## Data Availability

The pseudonymized dataset used and/or analyzed during the current study is available from the corresponding author upon request.

## Abbreviations

CC: Contingency coefficient
CCCC: Charité comprehensive cancer center
CRC: Colorectal cancer
ICD: International classification of diseases
OPS: Operationen- und prozedurenschlüssel (German procedure classification)
RCT: Radio-chemotherapy
UICC: Union internationale contre le cancer
